# Could serum uric acid to HDL cholesterol ratio predict sacroiliitis?

**DOI:** 10.1371/journal.pone.0289624

**Published:** 2023-10-23

**Authors:** Melike Elif Kalfaoglu

**Affiliations:** Department of Radiology, Izzet Baysal Training and Research Hospital, Bolu Abant Izzet Baysal University, Bolu, Turkey; UNITED KINGDOM

## Abstract

Recently, several inflammatory markers, including the uric acid to HDL cholesterol ratio (UHR), triglyceride/HDL cholesterol ratio (THR), systemic inflammatory index (SII), and C-reactive protein to albumin ratio (CAR), have been reported to be associated with inflammatory conditions. However, their collective role in sacroiliitis has not been extensively studied. This study aims to investigate the general characteristics and inflammatory markers in patients with and without sacroiliitis, and to observe any differences in these parameters in subjects with active and chronic sacroiliitis. Patient with sacroiliitis who showed up in the Radiology Department of Abant Izzet Baysal University Hospital were enrolled. Patients diagnosed with sacroiliitis based on clinical symptoms, physical examination, and conventional radiography or MRI findings were included in the sacroiliitis group. Patients without sacroiliitis who present with back pain or hip pain but have normal radiographic findings were included in the control group. General characteristics, including age, sex, body mass index (BMI), medical history, and disease duration, were collected from all participants. Blood samples were collected to measure inflammatory markers, including UHR, THR, SII, and CAR. The collected data were compared between sacroiliitis and control groups. Subgroup analysis was also performed to compare the inflammatory markers between subjects with active and chronic sacroiliitis. The median UHR of the sacroiliitis and control subjects were 11% (3–20%) and 7% (3–13%), respectively (p<0.001). Serum UHR was significantly and positively correlated with CRP (r: 0.4, p = 0.001) and ferritin (r: 0.17, p = 0.045) levels. In ROC analysis, a UHR level higher than 8% has an 81% sensitivity and 64% specificity in detecting sacroiliitis (AUC: 0.8, p<0.001, 95% CI: 0.72–0.84). In conclusion, we suggest that UHR could provide useful data as an additional diagnostic tool in patients with sacroiliitis.

## Introduction

Sacroiliitis is inflammation of sacroiliac joint, either bilaterally or unilaterally, and is a common cause of back pain. It can be seen in various conditions such as axial spondyloarthritis including ankylosing spondylitis or inflammatory arthritis. Common presenting symptoms of sacroiliitis may include uni/bilateral lower back pain or hip pain [1, 2].

Despite conventional radiography having low sensitivity and specificity in diagnosing sacroiliitis, it is still the first step in imaging studies. Conventional radiography cannot visualize the inflammatory phase when the structural changes have not initiated, but it can diagnose structural changes and ankyloses in the sacroiliac joint (SIJ) and classify them according to the modified New York Criteria (mNYC) [3]. Early inflammatory changes in the SIJ can be detected by magnetic resonance imaging (MRI), which is useful for both detecting early inflammatory lesions such as bone marrow edema, synovitis, and enthesitis, and late structural changes such as subchondral sclerosis, erosion, backfill/fat metaplasia, and ankyloses [4].

Novel inflammatory markers, including the uric acid to HDL cholesterol ratio (UHR), triglyceride/HDL cholesterol ratio (THR), systemic inflammatory index (SII) and C-reactive protein to albumin ratio (CAR), have been reported to be associated with inflammatory conditions. UHR is elevated in thyroiditis [5], metabolic syndrome [6], and diabetes mellitus type 2 [7]. Similarly, recent evidence suggests that THR can predict inflammatory diseases, such as hypertension [8], and type 2 diabetes [9]. Inflammatory diseases including type 2 DM [10], and infections [11], are characterized by elevated serum levels of CAR. However, to the best of our knowledge, these inflammatory markers have not been collectively studied in sacroiliitis. Therefore, we aimed to study the general characteristics and inflammatory markers (SII, CAR, THR, and UHR) in patients with and without sacroiliitis. We also aimed to observe differences in those parameters in subjects with active and chronic sacroiliitis.

## Methods

### Study population

Present retrospective cross sectional study was conducted in the radiology department of Abant Izzet Baysal University Hospital after obtaining ethical approval (Abant Izzet Baysal University Ethics Committee, date: 23^rd^ May, 2023 & approval no: 2023/122). After obtaining approval from the ethics committee, patient data was assessed from May 24 to 26, 2023. Patients with sacroiliitis who visited our institution between January 2021 to February 2023 were enrolled in the study. Control subjects were volunteers whose radiological imaging studies revealed a normal sacroiliac joint. We further grouped sacroiliitis patients according to the lesion characteristics either as active or chronic sacroiliitis.

Age, gender, and laboratory characteristics such as the erythrocyte sedimentation rate (ESR), C-reactive protein (CRP), aspartate and alanine transaminases (AST and ALT), ferritin, serum albumin, blood urea, creatinine, serum uric acid, triglyceride, HDL-cholesterol, leukocyte count (WBC), hemoglobin (Hb), hematocrit (Htc) and platelet count (Plt) were recorded from patients’ files and the institutional database. UHR was calculated by simply dividing the serum uric acid by the HDL cholesterol. Similarly, THR was calculated by dividing the triglyceride by the HDL cholesterol. SII was calculated using the following formula: (neutrophil count x Plt) / lymphocyte count. CAR was calculated by the following equation: CAR = CRP/serum albumin. Characteristics and laboratory data of the study groups were compared.

### Statistical analyses

Statistical software (SPSS 18 for Windows, IBM Co, Chicago, IL, USA) was used for the statistical analyses. The Kolmogorov-Smirnov test was applied to the study variables to assess normality. Variables that fit a normal distribution were analyzed using independent samples t-test and expressed as means and standard deviations. Other variables that did not fit a normal distribution were expressed as median (min-max) and compared with the Mann-Whitney U test. Categorical variables were compared between study groups using the chi-square test and presented as numbers and percentages. The correlation between study variables was analyzed using Pearson’s correlation test. The specificity and sensitivity of study parameters in detecting sacroiliitis were analyzed using ROC analysis test. Results were considered significant when the p value was less than 0.05%.

## Results

The study population icluded 129 subjects; 79 in the sacroiliitis group and 50 in the control group. The mean ages of the sacroiliitis and control groups were 39,4 ± 11,8 years and 41,2 ± 11,4 years, respectively, with no statistically significant difference (p = 0.38). In the sacroiliitis group, 54 (68%) were women, compared to 36 (72%) in the control group; the difference in gender composition was not statistically significant (p = 0.66).

There were no statistically significant differences between the study and control groups in terms of urea (p = 0.49), creatinine (p = 0.6), WBC (p = 0.09), Hb (p = 0.77), Htc (p = 0.45), Plt (p = 0.23), ESR (p = 0.41), ALT (p = 0.13), ferritin (p = 0.18), albumin (p = 0.18) and SII (p = 0.73) levels.

Serum uric acid (p<0.001), HDL cholesterol (p = 0.001), CRP (p = 0.004), AST (p = 0.03) and triglyceride (p = 0.02) levels of the study and control groups were significantly different.

The median UHR of the sacroiliitis and control subjects was 11% (3–20%) and 7% (3–13%), respectively (p<0.001). Similarly, the median THR of the sacroiliitis group (2,48% (0,5–30%)) was significantly higher than that of the control group (1,5% (0,6–6,4%)) (p = 0.002).

Serum UHR was significantly and positively correlated with CRP (r: 0.4, p = 0.001) and ferritin (r: 0.17, p = 0.045) levels. On the other hand THR was correlated with only serum CRP levels (r: 0.21, p = 0.002). Additionally, CAR was positively correlated with UHR (r:0.19, p = 0.02) and ESR (r: 0.41, p<0.001) levels.

Serum CAR was significantly elevated in the sacroiliitis group (0,45%(0,02–10,73%)) compared to the control group (0,16% (0,02–5,76%)), (p = 0.005).

Table 1 summarizes the data of the study and control groups.

**Table 1:** Data of the sacroiliitis and control group.

		Sacroiliitis	Control	P
Gender	Women (n,%)	54 (68%)	36(72%)	0.66
	Men (n,%)	25(%32)	14(%28)	0.66
Age (years)		39,4 ± 11,8	41,2 ± 11,4	0.38
Median (min-max)				
Urea (mg/dL)		28 (15–41)	26(11–60)	0.49
Creatinine (mg/dL)		0,76 (0,3–1,3)	0,76(0,53–1,1)	0.6
WBC (k/mm^3^)		7,05 (3,18–12,45)	6,64 (4,02–10,80)	0.09
Hb (g/dL)		13,50 (10,20–17,40)	13,75 (11,10–16,30)	0.77
Htc (%)		41,4 (29,8–52,6)	41,5 (30,4–48,7)	0.45
Plt (/mm^3^)		282000 (90000–487000)	261000 (68000–551000)	0.23
ESR (mm/h)		18 (2–57)	16 (2–51)	0.41
ALT (U/L)		18 (6–61)	14,5 (7–68)	0.13
Ferritin (ng/mL)		38 (3,59–202)	27,5 (5,2–195,7)	0.18
Albumin (g/L)		48 (27–56)	46,5 (37–54)	0.18
Serum Uric Acid (mg/dL)		5,2 (2,1–8)	4,0 (1,2–6,5)	**<0.001**
HDL Cholesterol (mg/dL)		48,7 (7,4–94,3)	58,6 (35,4–86,6)	**0.001**
CRP (mg/L)		2,1 (0,1–44,7)	0,8 (0,1–24,2)	**0.004**
AST (U/L)		18 (10–36)	16 (10–50)	**0.03**
Triglyceride (mg/dL)		118 (47–331)	93,5 (39–326)	**0.02**
SII (%)		569 (78–2860)	539 (150–1997)	0.73
UHR (%)		11 (3–20)	7 (3–13)	**<0.001**
THR (%)		2,48 (0,5–30)	1,5 (0,6–6,4)	**0.002**
CAR (%)		0,45 (0,02–10,73)	0,16 (0,02–5,76)	**0.005**

**Fig 1:**
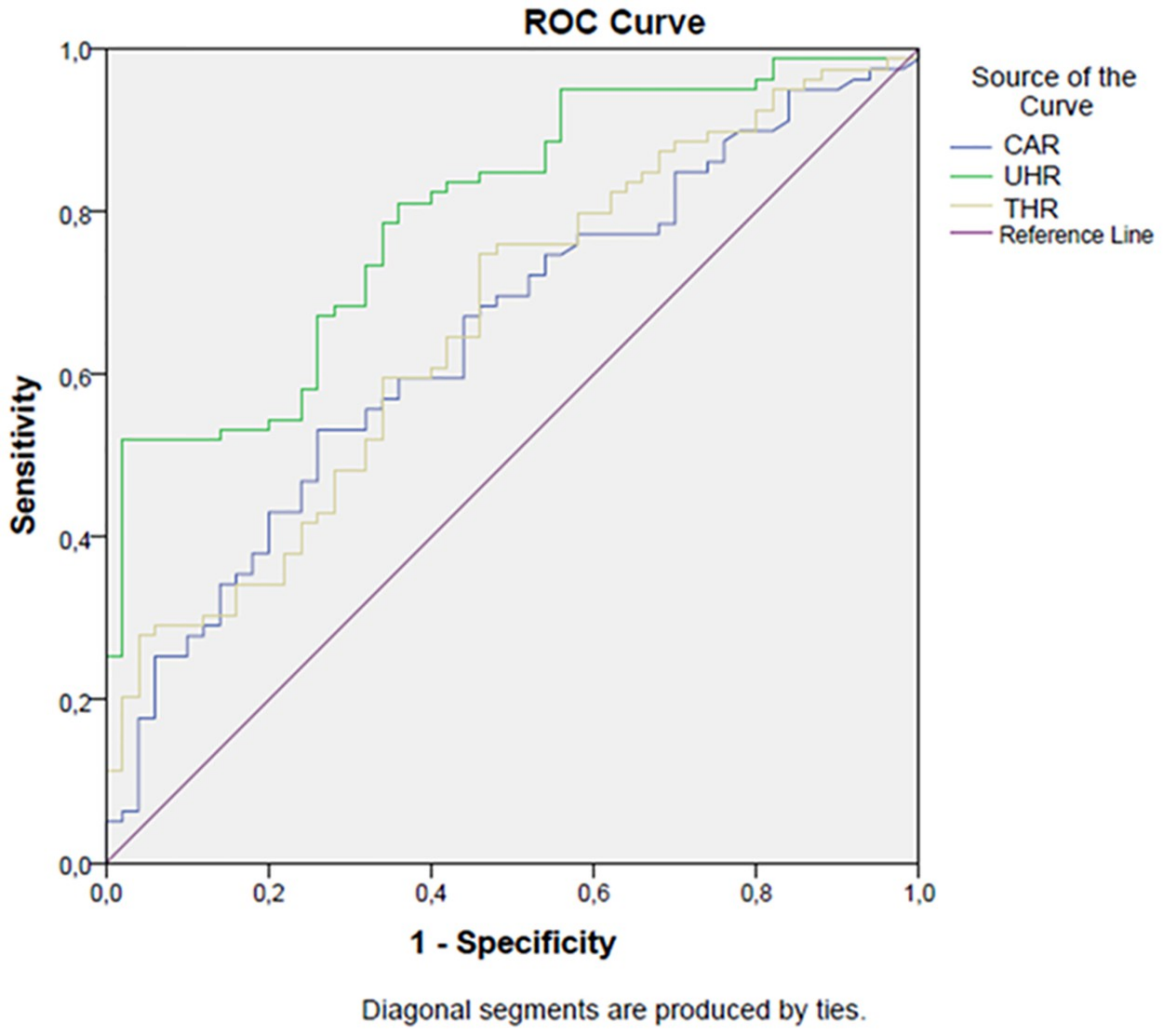
ROC analysis.

In ROC analysis, a UHR level higher than 8% has 81% sensitivity and 64% specificity in detecting sacroiliitis (AUC: 0.8, p<0.001, 95%CI: 0,72–0,84). Similarly a THR level higher than 1,6% has 75% sensitivity and 54% specificity in detecting sacroiliitis (AUC: 0.66, p = 0.048, 95%CI: 0,57–0,76). Additionally, a CAR level higher than 0,19% had 67% sensitivity and 56% specificity in detecting sacroiliitis. Fig 1 shows the ROC curve of the study parameters in detecting sacroiliitis.

In subgroup analysis, the data of the patients with active lesions and chronic lesions were compared. Serum albumin (p = 0.55), blood urea (p = 0.85), creatinine (p = 0.11), uric acid (p = 0.40), HDL cholesterol (p = 0.16), WBC (p = 0.52), Hb (p = 0.28), Htc (p = 0.56), Plt (p = 0.29), ESR (p = 0.97), CRP (p = 0.09), AST (p = 0.76), ALT (p = 0.85), ferritin (p = 0.08), triglyceride (p = 0.71), THR (p = 0.50), and SII (p = 0.22) values were not significantly different among sacroiliitis patients with active lesions and the subjects with chronic lesions.

On the other hand, the mean age (p = 0.02), median UHR (p = 0.002) and median CAR (p = 0.03) levels of the sacroiliitis patients with active lesions and the subjects with chronic lesions were significantly different.

## Discussion

The present work outlines that UHR, CAR and THR can serve as markers of sacroiliitis since these parameters are increased in patients with sacroiliitis. Moreover, CAR and UHR can also be useful in distinguishing sacroiliitis subjects with active lesions from those with chronic lesions. Moreover, both UHR, CAR and THR were significantly correlated with each other and other well-known inflammatory markers such as CRP and ESR. Finally, UHR, THR and CAR had considerably high sensitivity and specificity in detecting sacroiliitis (highest sensitivity and specificity for UHR).

Sacroiliitis has been reported to be characterized by an increase in serum levels of various inflammatory markers. For instance, the authors found increased high sensitivity CRP levels in patients with sacroiliitis confirmed by MRI compared to the controls [12]. Moreover, Jee et al. concluded that ESR and CRP were among the inflammatory markers associated with sacroiliitis [13]. Similarly, interleukin-7 was suggested as another inflammatory marker for patients with sacroiliitis [14]. Indeed, we found elevated levels of THR, UHR and CAR, which are introduced as novel markers of inflammation, in patients with sacroiliitis compared to the control subjects.

Recent evidence in the medical literature suggests that UHR can serve as a marker of chronic, low-grade inflammation in many situations. These include hypertension [15], fatty liver disease [16], coronary heart disease [17], and chronic renal failure [18]. Overt or subtle inflammation is a hallmark of all of these conditions. Accordingly, we found higher UHR levels in patients with sacroiliitis compared to healthy individuals in the present study.

Similar to the UHR, THR has also attracted great attention as a marker of inflammation, recently. For example, Chen et al. reported that THR could serve as an independent risk factor for cardiovascular events in the general population [19].

Additionally, increased THR has been reported to be associated with type 2 DM in several studies [20, 21]. Interestingly, elevated THR was correlated with blood pressures in hypertensive patients according to the Kurtkulagi et al’s study [8]. Metabolic diseases, such as hepatosteatosis, is also characterized with elevated levels of THR [22]. These conditions are associated with an increased inflammatory burden as is sacroiliitis. Therefore, we found elevated THR levels in sacroiliitis patients compared to the controls. Moreover, UHR had the greatest sensitivity and specificity in detecting patients with sacroiliitis, compared to other parameters.

Another actual inflammatory predictor is CAR which has been studied in many conditions. These include infectious diseases such as abscess [23], pneumonia [24], and Covid-19 disease [25]. Moreover, elevated CAR levels have been linked to various types of cancer [26–28], hypertension [29], inflammatory bowel disease [30], and diabetic nephropathy [10]. Similar to sacroiliitis, these conditions are also associated with inflammation. Thus, we found elevated levels of CAR in sacroiliitis subjects compared to healthy individuals. The present study showed that UHR, CAR and THR could be useful diagnostic tools along with radiological studies in the diagnosis and follow up of subjects with sacroiliitis.

The limitations of the present study may include its retrospective design, single center nature and relatively small study cohort. However, to the best of our knowledge, the present study is the first in the literature to observe novel inflammatory markers; CAR, UHR, SII and THR, in the sacroiliitis population.

In conclusion, we suggest that UHR, CAR and THR could provide useful data as additional diagnostic tool in patients with sacroiliitis. These markers can be taken into account by physicians in establishing the diagnosis of sacroiliitis due to their inexpensive, reliable, and easy to assess nature.

## Supporting information

S1 Checklist STROBE statement—Checklist of items that should be included in reports of observational studies. (DOCX)
